# Deep brain stimulation in posterior subthalamic area for Holmes tremor: Case reports with review of the literature

**DOI:** 10.3389/fneur.2023.1139477

**Published:** 2023-03-23

**Authors:** Hikaru Kamo, Genko Oyama, Masanobu Ito, Hirokazu Iwamuro, Atsushi Umemura, Nobutaka Hattori

**Affiliations:** ^1^Department of Neurology, Juntendo University School of Medicine, Tokyo, Japan; ^2^Department of Neurodegenerative and Demented Disorders, Juntendo University Graduate School of Medicine, Tokyo, Japan; ^3^Department of Home Medical Care System Based on Information and Communication Technology, Juntendo University Graduate School of Medicine, Tokyo, Japan; ^4^Department of Drug Development for Parkinson's Disease, Juntendo University Graduate School of Medicine, Tokyo, Japan; ^5^Department of PRO-Based Integrated Data Analysis in Neurological Disorders, Juntendo University Graduate School of Medicine, Tokyo, Japan; ^6^Department of Research and Therapeutics for Movement Disorders, Juntendo University Graduate School of Medicine, Tokyo, Japan; ^7^Department of Psychiatry, Juntendo University School of Medicine, Tokyo, Japan; ^8^Department of Neurosurgery, Juntendo University School of Medicine, Tokyo, Japan; ^9^Research and Therapeutics for Movement Disorders, Juntendo University Graduate School of Medicine, Tokyo, Japan; ^10^Neurodegenerative Disorders Collaborative Laboratory, RIKEN Center for Brain Science, Wako, Japan

**Keywords:** deep brain stimulation, posterior subthalamic area, cortico-basal ganglia loops, cerebellothalamic tract, Holmes tremor (HT)

## Abstract

**Background:**

Holmes tremor (HT) is a refractory tremor associated with cortico-basal ganglia loops and cerebellothalamic tract abnormalities. Various drug treatments have been attempted; however, no treatment method has yet been established. Historically, thalamic deep brain stimulation (DBS) has been performed in medically refractory cases. Recently, the posterior subthalamic area (PSA) has been used for HT. Here, we report cases of HT and review the effectiveness and safety of PSA-DBS for HT.

**Cases:**

We conducted a retrospective chart review of two patients with HT who underwent PSA-DBS. Improvement in tremors was observed 1 year after surgery without apparent complications.

**Literature review:**

We identified 12 patients who underwent PSA-DBS for HT, including our cases. In six patients, PSA was targeted alone; for the rest, the ventralis intermediate nucleus (Vim) of the thalamus and PSA were simultaneously targeted. The Fahn–Tolosa–Marin Tremor Rating Scale improvement rates were 56.8% (range, 33.9–82.1%; *n* = 6) and 77.8% (range, 42.6–100%; *n* = 5) for the PSA-DBS and PSA+Vim-DBS, respectively.

**Conclusion:**

Reasonable improvements in HT were observed after PSA-DBS. PSA might be an appropriate target for improving the symptoms of HT. Long-term observations, accumulation of cases, and randomized studies are required in future.

## Background

Holmes tremor (HT) is a slow below-4.5-Hz tremor observed during rest, action, and posture and was first reported by Holmes (1). HT is clinically defined in the recent consensus of the Movement Disorder Society as the presence of resting, postural, and intention tremors with tremor frequency below 4.5 Hz and onset with a variable delay between lesion occurrence and the first appearance of symptoms (2). The cortico-basal ganglia (BG) loop and the cerebellothalamic tract are suspected anatomical causative sites (3, 4). It has various causes, including cerebrovascular accidents, trauma, demyelinating diseases, and malignant tumors (5). Various medical and surgical treatments, including deep brain stimulation (DBS), have been attempted, but effective methods have not yet been established (6). We report two HT cases and compare them with previous cases to evaluate the efficacy and safety of DBS of the posterior subthalamic area (PSA) for HT.

## Case reports

### Case 1

**Figure 1:**
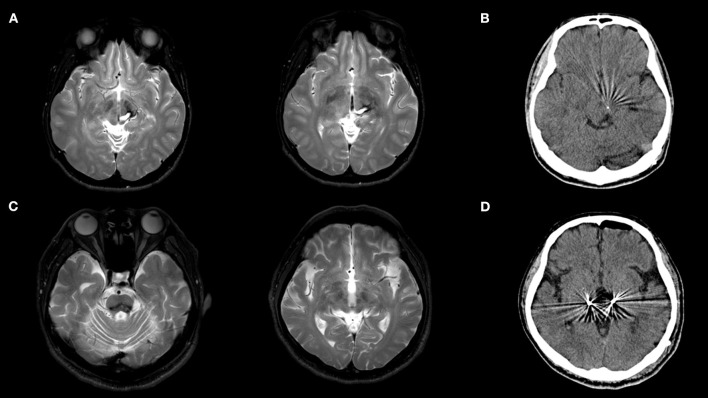
**(A)** Brain MRI showed an old hemorrhage in patient 1. **(B)** Postoperative CT showed the electrode was precisely implanted in the PSA in patient 1. **(C)** Brain MRI showed the old hemorrhage in the pons in patient 2. **(D)** Postoperative CT showed the electrodes were precisely implanted in the PSA in patient 2.

An 18-year-old man with a Korean father and a Japanese mother visited our hospital due to a 3-year history of right-hand tremors owing to a brain hemorrhage. The initial symptom was right-sided ataxic hemiparesis at the age of 15 years. He had no previous medical history, and the workup identified any cause of the hemorrhage. A right-sided tremor developed 3 months after the onset of the hemorrhage. He presented with a 4–5 Hz tremor in his right hand and lip during rest, posture, and movement. Brain magnetic resonance imaging (MRI) revealed an old hemorrhage from the posterior limb of the left internal capsule to the midbrain and left cerebellar peduncle and pseudohypertrophy of the inferior olivary nucleus (Figure 1A). A dopamine transporter scan [iodine-123 fluoropropyl carbomethoxy-3 beta-4-iodophenyltropane (^123^I-FP CIT)] revealed the absence of radiotracer activity in the left caudate and putamen. Clonazepam (0.5 mg/day), zonisamide (100 mg/day), and primidone (25 mg/day) were administered and showed a limited effect; however, these were discontinued owing to nausea. The levodopa challenge test (100 mg, intravenous) revealed no improvement in the Fahn–Tolosa–Marin Tremor Rating Scale (FTM-TRS) motor score, which was 62 (7). The patient decided to undergo DBS, and the left PSA was chosen as the target following an interdisciplinary team discussion. It was confirmed that the hemorrhage did not distort the anatomy of the target area (Figure 1A). Mapping and trajectory planning were performed with volumetric MRI using the Stealth Framelink^TM^ (Medtronic Japan) software. Permanent stimulating electrodes of the Vercise Cartesia^TM^ Directional Lead DBS system (Boston Scientific, USA) were implanted. The PSA target was identified using MRI as the white-matter region located outside the outermost edge of the red nucleus and posterior medial to the subthalamic nucleus (6 mm posterior, 5.5 mm inferior, and 8 mm lateral to the midcommissural (MC) point). Intraoperative macrostimulation showed that tremor was significantly improved by stimulus settings of (2–4)(–)Case(+), 2.0 mA, 60 μs, and 130 Hz. Subsequently, Vercise Genus R16 IPG^TM^ (Boston Scientific, USA) was implanted in the chest under general anesthesia. Brain CT revealed that the electrode was correctly placed in the target area, and analysis of the postoperative CT fused with preoperative MRI showed that the tip of the lead was at the coordinates of 6.8 mm posterior, 7.6 mm inferior, and 7.0 mm lateral to the MC point (Figure 1B). The patient presented with no postoperative side effects and showed improvement in his right arm tremors at rest, posture, and movement, even in the stimulation-off state. A monopolar review was performed when the stimulation was initiated 1 week after surgery, and it revealed that contacts 2–4 were the best contacts. Then, the horizontal directional steering found that the stimulation of contacts 4 and 7 worsened dysarthria, whereas stimulation of contacts 2 and 5 improved dysarthria. Then, stimulation was initiated with the following settings: (2–4)(–)Case(+), 0.5 mA, 60 μs, and 130 Hz. The tremor was further improved, and at 1-month evaluation post-surgery, the FTM-TRS motor score was 37 with increasing stimulation amplitude to 1.6 mA. Subsequently, the stimulation frequency was increased to 170 Hz, owing to insufficient efficacy, which was inferred to be caused by the decrease in the micro-lesioning effect over time. As the therapeutic effect was satisfactory, the stimulation intensity was reduced to 1.2 mA. At the 1-year evaluation after surgery, the motor score of FTM-TRS improved to 27 with stimulation settings of (2–4) (–) Case (+), 1.2 mA, 60 μs, and 170 Hz. After the 1-year evaluation, the horizontal directional steering was applied with stimulation settings of (2: 80%, 3:20%, 4:20%) (–) Case (+), 1.2 mA, 60 μs, and 170 Hz because the patient complained of dysarthria.

### Case 2

A 52-year-old Japanese man was referred to our hospital because of action and resting tremor in his right hand, which developed 1 year after the onset of right-sided hemiparesis and dysarthria due to left hypertensive cerebral hemorrhage at the age of 49. The tremor gradually worsened, and palatal and pharyngeal tremors developed. Levodopa/benserazide (450 mg/75 mg/day) and clonazepam (1 mg/day) were previously tested but showed minimal improvement in symptoms. He presented with a slow 4–5 Hz tremor on his soft palate, pharynx, and left-dominant upper and lower limbs during rest, posture, and movement. Brain MRI revealed an old hemorrhage from the midbrain to the pons and pseudohypertrophy of the inferior olivary nucleus (Figure 1C). A dopamine transporter scan (^123^ I-FP-CIT) revealed the absence of radiotracer activity in the left caudate and putamen. Zonisamide (200 mg/day) and primidone (250 mg/day) were initiated and showed limited improvement and they were discontinued owing to somnolence. The patient decided to undergo DBS for bilateral PSA. At that time, the motor score of the FTM-TRS was 56. The Vercise Cartesia^TM^ Directional Lead DBS system was implanted into the target. The PSA target was defined using MRI similar to case 1, and the coordination of the target was 7.5 mm posterior, 5.5 mm inferior, and 10.75 mm lateral to the MC point on the left side and 7 mm posterior, 5.5 mm inferior, and 11 mm lateral to the MC point on the right side. Intraoperative macrostimulation revealed that tremor was improved by a stimulus of (5–7)(–)Case(+), 3.0 mA, 60 μs, and 130 Hz on both sides. After the implantation of the DBS lead, Vercise Genus R16 IPG^TM^ (Boston Scientific, USA) was implanted in the chest under general anesthesia. Brain CT revealed that the electrode was placed in the target area, and analysis of the postoperative CT fused with preoperative MRI showed that the tip of the lead was at the coordinates of 8.1 mm posterior, 7.2 mm inferior, and 10.0 mm lateral in right and 8.2 mm posterior, 7.3 mm inferior, and 10.0 mm lateral in left to the MC point (Figure 1D). Stimulation was started 1-week after surgery, and with increasing stimuli, the upturning of the eyeball was noted as a side effect. Therefore, directional stimulation was used to prevent oculomotor involvement. The stimulation settings were adjusted as follows: 1(–)8(+), 4.0 mA, 50 μs, and 185 Hz on the left side; and 1(–)8(+), 6.5 mA, 40 μs, and 185 Hz on the right side, and zonisamide was reduced to 100 mg/day. At 1-month postoperative evaluation, a motor score of FTM-TRS score was 44 and the pulse width was increased to 50 μs on the right side, owing to inadequate effectiveness. At the 1-year postoperative evaluation, the motor score of FTM-TRS remained at 37 without side effect.

## Literature review

To investigate the relationship between HT and PSA-DBS, we reviewed published scientific reports using the PubMed database. The keywords used were “Holmes tremor,” “Rubral tremor,” or “midbrain tremor,” “deep brain stimulation,” and “posterior subthalamic area” or “caudal zona incerta.” In addition, the references of the included articles were screened for eligible studies. Only studies published in English between 2006 and 2021 that examined the association between HT and PSA-DBS were reviewed. Of the four records identified, three were included in the review. Two additional articles were identified by screening their references. A total of five studies along with our study were included in the review (Table 1) (8–12). We identified 12 patients who underwent PSA-DBS, including our patients. In six patients, PSA was targeted alone, and in the rest, both ventralis intermediate nucleus (Vim) and PSA were targeted. The period from disease onset to surgery was 1–39 years old. The mean age at the time of surgery was 18–84 years. The average FTM-TRS improvement rates were 56.8% (range, 33.9–82.1%; *n* = 6) in the PSA group and 77.8% (range, 42.6–100%; *n* = 5) in the PSA+Vim group.

**Table 1:** Clinical outcomes of PSA-DBS for HT.

**References**	**Pt**	**Sex**	**Etiology of HT**	**Duration of HT, y**	**Age at DBS, y**	**DBS target**	**FTM-TRS motor score**				**SE**
							**Pre-DBS**	**1 m after DBS**	**12 m after DBS**	**Imp 12 m after DBS, %**	
Our cases	1	M	CH	6	21	Lt PSA	62	37	27	56.4	Dysarthria
	2	M	CH	1	52	Bil PSA	56	44	37	33.9	Upturning of the eyeball
Dec-Cwiek et al. (8)	1	M	MS	10	38	Lt PSA + Bil Vim	54	-	31	42.6	Dysarthria
	2	F	CI	39	50	Lt PSA	39	-	7	82.1	None
	3	M	CI	1	48	Lt PSA	36	-	15	58.3	None
Yuk et al. (9)	1	M	CH	1	55	Rt PSA	47	39	30	36.1	None
Kobayashi et al. (10)	1	F	CH/tumor	6	19	Rt PSA + Vim	12	-	0	100	None
	2	M	CI	3	67	Lt PSA + Vim	17	-	3	82.4	None
	2	M	CH	1	44	Rt PSA + Vim	22	-	5	77.2	None
	3	M	Trauma	2	18	Rt PSA + Vim	15	-	2	86.7	None
O'Shea et al. (11)	1	F	CI	1	62	Rt PSA + Vim	-	-	-	-	None
Plaha et al. (12)	1	M	-	6	84	Bil PSA	50	-	13	74	Dysphasia

We have identified 12 patients with PSA-DBS, including our cases. Six cases targeted PSA alone, and five targeted both Vim and PSA. Pt, patient; HT, Holmes tremor; DBS, deep brain stimulation; y, year; m, month; imp, improvement; SE, side effect, CH, cerebral hemorrhage, CI, cerebral infarction; MS, multiple sclerosis; M, male; F, female; Lt, left; Rt, right; Bil, bilateral; PSA, posterior subthalamic area; Vim, thalamic ventral intermediate.

## Discussion

Historically, Vim has been reported as a target for HT in stereotactic surgery (13–22). Subsequently, other targets, such as ventralis oralis posterior (14), ventralis oralis anterior (23), prelemniscal radiation (24), pallidotomy (25, 26), subthalamic nucleus (4), caudal zona incerta (Zi) (12), and globus pallidus internus (GPi) (27–29), have been reported. A previous systematic review compared GPi-DBS (*n* = 21) and Vim-DBS (*n* = 37) for HT and reported that GPi-DBS was more effective in suppressing tremors. Moreover, the study also compared the benefits among patients treated with multiple targets (two or three) vs. a single target; however, no significant difference was noted. It was speculated that GPi-DBS provides more robust tremor suppression because of stimulation involved in the BG-thalamocortical circuit (30). In this context, the stimulation of the ventralis oralis may be effective in suppressing the pallidal receiving area (15, 31). However, the best DBS target for HT has not been established due to the small number of cases. Table 2 shows the available literature on previous stereotactic targets for HT. The variability of the study period and the evaluation methods among studies made it difficult to directly compare the effect of each target. Recently, PSA has emerged as an alternative target for tremor treatment (32–34). The PSA is located anterior to the medial colliculus, lateral to the red nucleus, and posterior to the subthalamic nucleus and includes the Zi. PSA involves the pallidothalamic and cerebellar-thalamic tracts, descending fibers, and dentate-rubro-thalamic tract (DRTT) (35). PSA-DBS directly affects DRTT and pallidothalamic tract and has effects on tremors (36). A randomized, double-blind study confirmed that no differences between Vim and PSA related to the side effects of stimulation exist and that PSA-DBS can achieve the same level of efficacy with a lower stimulation amplitude in essential tremors (37). Several studies have reported PSA as a DBS target for HT (8–12). In some cases, PSA is chosen as an additional target, in addition to Vim. Dec-Cwiek et al. reported PSA-DBS performed in three patients with HT. The Vim+left PSA was selected as the target in one patient, and only the left PSA was selected in two patients. After 12 months of follow-up, the bilateral Vim+left PSA case showed a 42.6% improvement in motor score of FTM-TRS compared to the baseline, while left PSA cases showed an improvement of 82.1 and 58.3%. The left PSA+bilateral Vim cases developed dysarthria as a stimulus-induced side effect (8). Yuk et al. reported a case of right-sided PSA-DBS in a patient with HT with a 36.1% improvement in motor score of FTM-TRS (9). O'Shea et al. reported a case of right-sided PSA+Vim-DBS for HT; however, both the frequency and amplitude of tremor improved 2 weeks after surgery (11). Plaha et al. reported a case of bilateral PSA-DBS for HT with a 74.0% improvement in motor score of FTM-TRS at 1 year after surgery (12). Kobayashi et al. reported the efficacy of dual electrodes inserted into the Vim and PSA of four patients with HT. In one patient, the tremor disappeared after DBS surgery, and no symptoms were observed even when DBS was turned off; therefore, a comparison could not be made. The improvement rate of the motor score of FTM-TRS was 86.5% (range, 77–100%) 1 year after surgery. When Vim-DBS and PSA-DBS were compared, the improvement rate of the motor score of FTM-TRS was 62.3% (range, 50–77%) when only the Vim was stimulated and 63.3% (range, 40–100%) when only PSA was stimulated, with no significant difference between the two groups. However, when the Vim and PSA were stimulated simultaneously, the improvement rate was 93.3% (range, 77–100%), showing a predominant increase in the improvement rate. The authors concluded that the stimulation of both targets, instead of PSA or Vim alone, is important (10). Based on the cases from previous literature and our cases, the improvement rates in the motor score of FTM-TRS were 56.8% (range, 33.9–82.1%; *n* = 6) with the PSA and 77.8% (range, 42.6–100 %; *n* = 5) with the PSA+Vim.

**Table 2:** Clinical outcomes of other DBS targets for HT.

**References**	**No. of pt**	**Scale**	**DBS target**	**Improvement**	**SE**	**FU period**
Foote and Okun (14)	1	FTM-TRS and TDS	Uni Vim	TRS 37%, TDS 80%	None	1 y
Foote et al. (15)	3	FTM-TRS	Uni Vim	51.15% (38.46–66.67)	None	6–12 m
Diederich et al. (16)	2	CGI-global improvement	Uni Vim	Moderate improvement	None	5–7 y
Sanborn et al. (17)	1	CGI-global improvement	Uni Vim	Full tremor suppression	None	2 y
Acar et al. (18)	1	CGI-global improvement	Bil Vim	Moderate improvement	None	3 y
Follett et al. (19)	1	TETRAS	Bil Vim	Significant tremor reduction	None	1 y
Issar et al. (20)	5	FTM-TRS	3 Uni Vim, 1 bil Vim, 1 bil GPi	14–36% in 3 uni Vim	Dystonia in UE, ataxia	2–3 y in 3 uni Vim
Romanelli et al. (4)	1		Uni Vim + STN	Full tremor suppression	None	2 y
Nikkhah et al. (21)	1		Uni Vim	Full tremor suppression	Facial parethesia	6 m
Goto and Yamada (26)	1		Uni Vim DBS and Pallidotomy	Full tremor suppression	None	18 m
Peker et al. (23)	1		Uni Vim, Voa and GPi	Full tremor suppression	None	1.5 y
Martinez et al. (28)	10	FTM-TRS	2 uni Vim, 1 bil Vim, 6 uni GPi, 1 bil GPi	Rest 87.25% (80–100, *n* = 9), posture 100% (*n* =1), intention 68.89% (55–80, *n* = 9)		2–12 y
Kilbane et al. (29)	4	FTM-TRS	Uni GPi	78.87% (59.9–94.4)	Dysarthria, ataxia	18–52 m
Franzini et al. (22)	9		6 uniVim, 3 bil Vim	>50% reduction in all cases	Dysarthria in 3 bil Vim	8 y
Martinez et al. (24)	1		Uni Raprl	Significant tremor reduction		2 y

No, number; Pt, patient; DBS, deep brain stimulation; FU, follow-up; SE, side effect; FTM-TRS, Fahn–Tolosa–Marin Tremor Rating Scale; Bil, bilateral; y, years; Uni, unilateral; Vim, ventral intermediate nucleus; TDS, tremor disability scores; m, month; CGI, Clinical Global Impression; TETRAS, Tremor Rating Assessment Performance Subscale; Voa, ventralis oralis anterior; Raprl, prelemniscal radiation.

Although the exact mechanism of HT is not known, both the cerebellothalamic pathway and the BG-thalamocortical circuit are thought to contribute to the pathophysiology of HT. PSA may be a relatively versatile target for stimulation that can further stimulate multiple circuits with fewer stimuli because PSA is the site where multiple pathways pass.

Regarding the side effect of PSA-DBS, Barbe et al. reported three events of ataxic gait and four events of dysarthria among 15 patients in a randomized, double-blind, crossover trial (37). The literature review shows similar results (Tables 1, 2). These are similar to Vim-DBS that is estimated to be induced by the stimulation of the same cerebellar connections (37). Owing to their anatomical location, dysarthria and ataxia have been reported as stimulus-induced side effects (37–39). Indeed, in our case, dysarthria was observed but these side effects were successfully managed using bipolar configuration or horizontal directional steering. Therefore, studies of the methods for detecting the more selective and precise target location for PSA-DBS, such as tractography, should be needed.

Our study revealed the efficacy and feasibility of PSA-DBS for HT. There is insufficient evidence to determine the best target among PSA, Vim, and GPi, and whether single or multiple targets should be stimulated for HT. Further prospective trials are required.

## Data availability statement

The datasets presented in this article are not readily available because of ethical and privacy restrictions. Requests to access the datasets should be directed to the corresponding author.

## Ethics statement

The studies involving human participants were reviewed and approved by the Ethics Committee of Juntendo University School of Medicine. The patients provided their written informed consent to participate in this study. Written informed consent was obtained from the individuals to publish any potentially identifiable images or data in this article.

## Author contributions

HK: conception, organization, execution (research project), and writing of the first draft (manuscript preparation). GO, MI, HI, and AU: conception, organization, execution (research project), and review and critique (manuscript preparation). NH: conception, execution (research project), and review and critique (manuscript preparation). All authors contributed to the article and approved the submitted version.
